# Pregnane X Receptor Activation in Liver Macrophages Protects against Endotoxin‐Induced Liver Injury

**DOI:** 10.1002/advs.202308771

**Published:** 2024-03-13

**Authors:** Tingting Zhao, Guoping Zhong, Ying Wang, Renjie Cao, Shaofei Song, Yuan Li, Guohui Wan, Haiyan Sun, Min Huang, Huichang Bi, Yiming Jiang

**Affiliations:** ^1^ Guangdong Provincial Key Laboratory of New Drug Design and Evaluation School of Pharmaceutical Sciences Sun Yat‐Sen University Guangzhou 510006 China; ^2^ Institute of Clinical Pharmacology Sun Yat‐Sen University Guangzhou 510006 China; ^3^ Sun Yat‐Sen Memorial Hospital Sun Yat‐Sen University Guangzhou 510006 China; ^4^ School of Food and Drug Shenzhen Polytechnic University Shenzhen 518055 China; ^5^ NMPA Key Laboratory for Research and Evaluation of Drug Metabolism & Guangdong Provincial Key Laboratory of New Drug Screening School of Pharmaceutical Sciences Southern Medical University Guangzhou 510006 China

**Keywords:** endotoxin, liver injury, macrophage polarization, pregnane X receptor

## Abstract

Endotoxemia‐related acute liver injury has a poor prognosis and high mortality, and macrophage polarization plays a central role in the pathological process. Pregnane X receptor (PXR) serves as a nuclear receptor and xenosensor, safeguarding the liver from toxic stimuli. However, the effect and underlying mechanism of PXR activation on endotoxemic liver injury remain largely unknown. Here, the expression of PXR is reported in human and murine macrophages, and PXR activation modified immunotypes of macrophages. Moreover, PXR activation significantly attenuated endotoxemic liver injury and promoted macrophage M2 polarization. Macrophage depletion by GdCl_3_ confirmed the essential of macrophages in the beneficial effects observed with PXR activation. The role of PXR in macrophages is further validated using AAV8‐*F4/80*‐*Pxr* shRNA‐treated mice; the PXR‐mediated hepatoprotection is impaired, and M2 polarization enhancement is blunted. Additionally, treatment with PXR agonists inhibited lipopolysaccharide (LPS)‐induced M1 polarization and favored M2 polarization in BMDM, Raw264.7, and THP‐1 cells. Further analyses revealed an interaction between PXR and p‐STAT6 in vivo and in vitro. Moreover, blocking *Pxr* or *Stat6* abolished the PXR‐induced polarization shift. Collectively, macrophage PXR activation attenuated endotoxin‐induced liver injury and regulated macrophage polarization through the STAT6 signaling pathway, which provided a potential therapeutic target for managing endotoxemic liver injury.

## Graphical abstract

The present study uncovers a novel role of pregnane X receptor (PXR) in inflammation‐mediated damage. PXR activation in macrophages inhibits M1 polarization and favors M2 polarization by interacting with phosphorylated signal transducer and activator of transduction 6 (p‐STAT6), which protects against endotoxin‐induced liver injury. PXR may be highlighted as a candidate therapeutic target for managing endotoxemic liver injury.

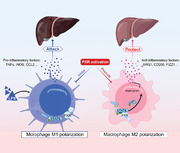

## 1 Introduction

Endotoxemia, a lethal disease caused by the release of endotoxin into the blood, manifests as systemic and excessive inflammation. Lipopolysaccharide (LPS), a main component of the outer membrane of gram‐negative bacteria, is a well‐known endotoxin.^[^ ^1^ ^]^ LPS stimulates immune cells to secrete a spectrum of pro‐inflammatory cytokines and chemokines, inducing inflammatory cascades that lead to an immune system imbalance.^[^ ^2^ ^]^ Severe endotoxemia can result in multi‐organ failure and septic shock, which are associated with a high mortality rate and poor prognosis.^[^ ^3^ ^]^ Serving as the primary immune barrier against pathogenic microorganisms, the liver becomes a target in endotoxemia. Acute liver injury is a clinical hallmark of endotoxemia and often signals the onset of septic organ failure.^[^ ^4^ ^]^

Hepatic macrophage populations consist primarily of monocyte‐derived and tissue‐resident macrophages.^[^ ^5^ ^]^ Kupffer cells are the resident macrophages in the liver, accounting for 80% of the total macrophage population in the human body.^[^ ^6^ ^]^ Macrophages are heterogeneous and highly plastic cells that polarize into distinct states in response to specific stimuli. The classical M1 phenotype is induced by LPS, which elicits the secretion of various inflammatory mediators, including tumor necrosis factor α (TNFα), interleukin 6 (IL6) and nitric oxide (NO), to counteract pathogenic microbial invasion.^[^ ^3^, ^7^ ^]^ While the M1 phenotypes play a crucial role in eliminating bacterial pathogens, prolonged M1 polarization can lead to a hyperinflammatory state that causes liver damage. In contrast, Kupffer cells can be induced by IL4 or IL13 to polarize into the alternative M2 phenotype, which produces anti‐inflammatory or pro‐resolving mediators, such as IL10, arginase 1 (ARG1), mannose receptor C type 1 (CD206) and transglutaminase 2 (TGM2).^[^ ^8^ ^]^ M2 macrophages contribute to inflammation regression, tissue repair, and immune function regulation.^[^ ^9^ ^]^ Dysregulated hyperinflammation is the pathological basis of liver injury associated with endotoxemia, and macrophages have been considered the primary pathogenic immune cells implicated in this process.^[^ ^10^ ^]^ Given these detrimental consequences of endotoxin‐induced acute liver injury, it is urgently required to conduct in‐depth mechanistic studies to identify potential therapeutic targets. Recently, strategies to modulate macrophage polarization have emerged as promising therapeutic approaches for endotoxin‐induced liver injury.

Pregnane X receptor (PXR) is a nuclear receptor involved in the regulation of xenobiotic and endobiotic detoxification. Although PXR is primarily expressed in the liver and gastrointestinal tract, recent studies have identified its presence in immune cells, including T lymphocytes and B lymphocytes. In these immune cells, PXR is reported to regulate the innate immune response.^[^ ^11^ ^]^ A wide range of ligands can activate PXR, including the rifampicin (RIF), pregnenolone 16α carbonitrile (PCN), dexamethasone and the natural herb St. John's Wort.^[^ ^12^ ^]^ RIF and rifaximin, recognized as potent human PXR agonists, have demonstrated therapeutic efficacy in the treatment of autoimmune skin disease and inflammatory bowel disease, respectively.^[^ ^13^ ^]^ These findings suggest a promising therapeutic strategy associated with PXR in managing inflammatory or immune‐mediated diseases.

The signal transducer and activator of transduction 6 (STAT6) is a critical regulator of the innate immune response that suppresses inflammation and promotes M2 polarization in macrophages. Once activated, STAT6 is phosphorylated and transported to the nucleus where it regulates downstream polarization‐related genes, such as suppressor of cytokine signaling 1 (SOCS1).^[^ ^14^ ^]^ In addition, STAT6 coordinates with Krüppel‐like factor 4 (KLF4) and CCAAT/enhancer binding protein beta (C/EBPβ) to promote macrophage M2 polarization, thereby exerting anti‐inflammatory effects.^[^ ^8^, ^15^ ^]^ The inflammation‐suppressing efficacy of STAT6 has been verified in various pathologies, including acute lung injury, stroke and hepatic ischemia‐reperfusion.^[^ ^14^, ^16^ ^]^ Notably, previous reports have noted the capacity of the STAT6 pathway to alleviate LPS/D‐galactosamine (GaIn)‐induced acute liver injury by promoting macrophage M2 polarization.^[^ ^16^ ^]^

The present study aimed to explore the role of PXR in LPS/GaIn‐induced liver injury and determine the potential mechanisms. Our data showed a novel function for PXR in alleviating LPS/GaIn‐induced liver injury and promoting macrophage M2 polarization via the STAT6 signaling pathway. Specifically, PXR's presence in macrophages was essential to the protective effect of PXR activation against LPS/GaIn‐induced liver injury. These findings uncovered the role of PXR in macrophages and its beneficial effect on endotoxin‐induced liver injury and proposed a potentially relevant clinical strategy for leveraging PXR as a therapeutic target for a range of hyperinflammatory diseases.

## 2 Results

### 2.1 PXR is Expressed in Macrophages and Regulates the Polarization

**Figure 1:**
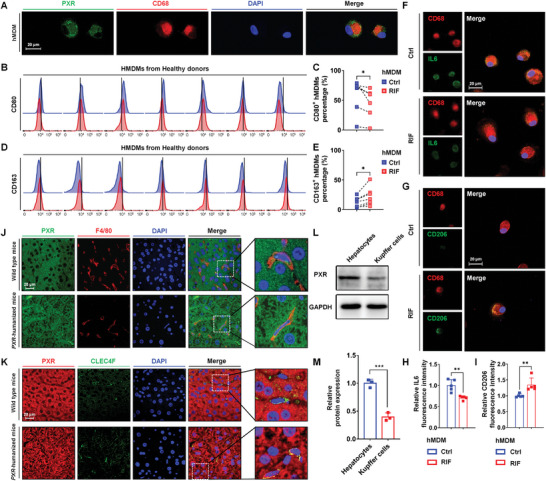
Expression and localization of PXR in human and murine macrophages. A) Immunofluorescence staining of hMDMs with PXR (green) and human macrophage marker CD68 (red). B–E) Flow cytometric histogram and proportion of CD80^+^ (M1) and CD163^+^ (M2) hMDMs incubated with DMSO or RIF (20 µm for 48 h) after isolation and differentiation from healthy donors (*n* = 7). ^*^ *p* < 0.05 as the RIF group compared with the control group using paired t‐test. F–I) Immunofluorescence staining and quantification of CD68 (red) and IL6 (green) or CD206 (green) in hMDMs incubated with DMSO or RIF (20 µM for 48 h) (*n* = 5). The data are expressed as the mean ± SD. ^**^ *p* < 0.01 as the RIF group compared with the control group. J, K) Immunofluorescence staining of liver sections from wild‐type and *PXR*‐humanized mice. J) PXR (green) and F4/80 (red) double staining, K) PXR (red) and CLEC4F (green) double staining. L,M) Western blotting and quantification of PXR expression in hepatocytes and Kupffer cells (*n* = 3). The data are expressed as the mean ± SD. ^***^ *p* < 0.001 as hepatocytes group compared with Kupffer cells group.

The identity of PXR in macrophages remained unclear, the presence and polarization‐regulating function of PXR in human monocyte‐derived macrophages (hMDMs) was investigated. hMDMs were isolated and differentiated from 12 healthy donors (Figure S1A,B, Supporting Information). Double immunofluorescence staining with PXR and human macrophage marker cluster of differentiation 68 (CD68) showed that PXR was predominantly enriched in the cytoplasm of hMDMs (**Figure** **1**A). Furthermore, the expression of macrophage membrane markers of M1 and M2 polarization was determined in the presence of RIF, a clinically human‐specific PXR agonist. Flow cytometry analysis revealed that PXR activation decreased the number of CD80^+^ hMDMs, while significantly increasing the number of CD163^+^ hMDMs, implying an inhibition of M1 polarization and promotion of M2 (Figure 1B–E). Additionally, immunofluorescence staining showed RIF significantly decreased the expression of IL6 (M1 marker) and enhanced the CD206 (M2 marker) expression in hMDMs (Figure 1F–I).

To extend our observations of PXR in macrophages, the localization and expression of PXR in macrophages were determined in murine hepatic macrophages and Kupffer cells. Immunofluorescence analysis of liver sections from wild‐type and *PXR*‐humanized mice revealed that adhesion G protein‐coupled receptor E1 isoform 3 precursor (F4/80, a marker for macrophages or Kupffer cells)‐positive and C‐type lectin domain family 4 member F (CLEC4F, a marker for Kupffer cells)‐positive cells expressed PXR, which was diffusely distributed throughout the macrophage (Figure 1J,K; Figure S1E, Supporting Information). Subsequently, PXR abundance in Kupffer cells and hepatocytes was determined by isolating these two types of cells from the mouse liver. Immunoblot analysis showed that the protein level of PXR in Kupffer cells was ≈39.4% of that in hepatocytes (Figure 1L,M). These results suggested that PXR was expressed in human and murine macrophages and had the potential to regulate the immunophenotype.

### 2.2 Activation of PXR Protects against LPS/GaIn‐Induced Liver Injury and Regulates Macrophage M1/M2 Polarization

**Figure 2:**
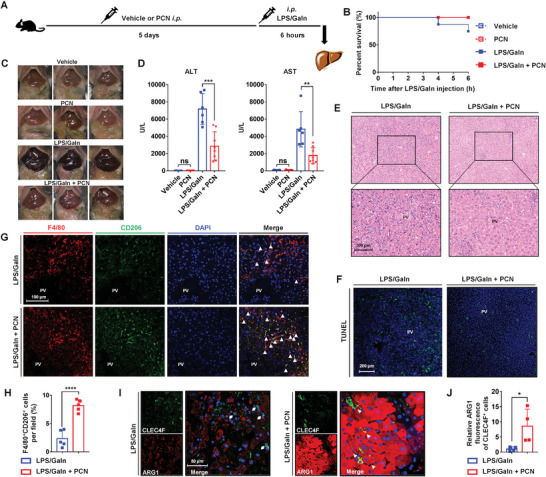
PXR activation protects against LPS/GaIn‐induced liver injury and promotes macrophage M2 polarization. A) Mice were treated with PCN (100 mg kg^−1^/d) or vehicle (corn oil) for 5 days, LPS/GaIn (100 µg kg^−1^ and 700 mg kg^−1^, respectively) was administered, and all mice have sacrificed 6 h post injection (*n* = 5 for vehicle and PCN group, *n* = 8 for LPS/GaIn and LPS/GaIn + PCN group). B) Survival curves for 6 h after injection. C) Representative gross pictures of the liver. D) Serum ALT and AST levels. E,F) Representative H&E and TUNEL staining of liver sections. G,H) Immunofluorescence staining and quantification of F4/80 (red) and CD206 (green) double staining (*n* = 5). Filled triangles indicate the F4/80^+^CD206 ^+^ macrophages. I,J) Immunofluorescence staining and quantification of CLEC4F (green) and ARG1 (red) double staining. Diamonds indicate CLEC4F^+^ARG1^−^ cells and filled triangles indicate CLEC4F^+^ARG1^+^ cells (*n* = 4). The data are expressed as the mean ± SD. ^*^ *p* < 0.05, ^**^ *p* < 0.01, ^***^ *p* < 0.001, and ^****^ *p* < 0.0001 compared with the LPS/GaIn group. *p* > 0.05 compared with the vehicle group, ns: not significant.

To investigate the effect of PXR on LPS/GaIn‐induced liver injury, mice were administered 100 mg kg^−1^/d PCN (a mouse‐specific PXR agonist) for 5 days and received a single intraperitoneal injection of LPS/GaIn (**Figure** **2**A). After PCN treatment for 5 days, hepatic PXR was successfully activated, as revealed by the expression of the downstream proteins UDP glucuronosyltransferase family 1 member A1 (UGT1A1), cytochrome P450 family 3 subfamily A polypeptide 11 (CYP3A11), and glutathione S‐transferase mu 2 (GSTM2) (Figure S2A,B, Supporting Information). LPS/GaIn treatment caused severe congestion in the liver and 25% mortality within 6 h. PXR activation markedly relieved liver congestion and improved survival rates (Figure 2B,C). To assess the pathological changes induced by LPS/GaIn, serum aminotransferases were examined, and hematoxylin and eosin (H&E) and terminal deoxynucleotidyl transferase dUTP nick end labeling (TUNEL) staining were performed. As expected, PCN treatment substantially decreased the LPS/GaIn‐induced high levels of serum alanine transaminase (ALT) and aspartate transaminase (AST) (Figure 2D). The H&E staining results showed multifocal hemorrhages around the portal vein (PV), remarkable distribution of liver architecture, and extensive necrosis in the livers of mice treated with LPS/GaIn, while the liver tissue structure of mice that were treated with PCN was intact, with neatly arranged hepatocytes (Figure 2E). And hepatocyte death was decreased, as determined by significantly reduced numbers of TUNEL^+^ cells in PCN‐treated livers (Figure 2F; Figure S2C, Supporting Information). These results demonstrated that PXR activation attenuated LPS/GaIn‐induced liver injury.

The polarization of macrophages plays a key role in LPS/GaIn‐induced liver injury. Therefore, we further investigated the polarization states of macrophages in the liver by double staining of F4/80‐induced nitric oxide synthase (iNOS) to show M1 polarization, and F4/80‐CD206 or CLEC4F‐ARG1 to show M2 polarization. Compared with the LPS/GaIn group, PXR activation significantly decreased the number of F4/80^+^iNOS^+^ macrophages and robustly increased the number of F4/80^+^CD206^+^ macrophages in the liver (Figure S2D,E, Supporting Information; Figure 2G,H). Similarly, the fluorescence intensity of ARG1 in Kupffer cells was dramatically increased by PXR activation, which was ≈8.6‐fold higher compared to the LPS/GaIn group (Figure 2I,J). On the other hand, PCN treatment markedly enhanced anti‐inflammation cytokine IL10 expression throughout the liver compared to the LPS/GaIn group (Figure S2G,H, Supporting Information). Additionally, Kupffer cells were isolated after liver perfusion, and the changes in M2 gene signatures in Kupffer cells were detected. As anticipated, PCN treatment significantly induced the expression of M2 markers, including *Il10*, macrophage galactose‐type C‐type lectin 1 (*Mgl1*), C‐type mannose receptor 2 (*Mrc2*), *Cd163*, found in inflammatory zone 1 (*Fizz1*) and transforming growth factor beta (*Tgfβ*) (Figure S2F, Supporting Information). These results showed that PXR activation inhibited M1 macrophage polarization and promoted M2 macrophage polarization.

### 2.3 PXR Activation Modulates Macrophage M1/M2 Polarization In Vitro

**Figure 3:**
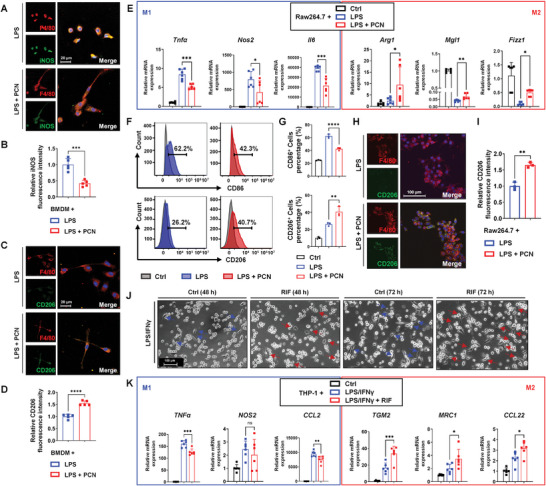
The regulatory effects of PXR on macrophage polarization of BMDMs, Raw264.7 and THP‐1 macrophages. BMDMs were incubated with LPS (1 µg mL^−1^, for 6 h) with or without PCN (20 µm, for 48 h). A,B) Immunofluorescence staining of F4/80 (red) and iNOS (green) in BMDMs and quantification of the iNOS fluorescence intensity (*n* = 5). C,D) Immunofluorescence staining of F4/80 (red) and CD206 (green) in BMDMs and quantification of the CD206 fluorescence intensity (*n* = 5). Raw264.7 cells were treated with vehicle or LPS (1 µg mL^−1^, for 24 h) with or without PCN (20 µm, for 48 h). E) Quantitative real‐time PCR (qRT‐PCR) analysis for M1 polarization‐related genes (*Tnfα*, *Nos2*, and *Il6*) and M2 polarization‐related genes (*Arg1*, *Mgl1*, and *Fizz1*) in Raw264.7 cells (*n* = 6). F,G) Histogram and proportion of CD86^+^ or CD206^+^ Raw264.7 macrophages by flow cytometry (*n* = 3). H,I) Immunofluorescence staining of F4/80 (red) and CD206 (green) in Raw264.7 macrophages and quantification of the CD206 fluorescence intensity (*n* = 3). ^*^ *p* < 0.05, ^**^ *p* < 0.01, ^***^ *p* < 0.001, and ^****^ *p* < 0.0001 compared with the LPS group. J) The morphology of THP‐1 macrophages with incubation of LPS/IFNγ with or without RIF (20 µm) for 48 and 72 h. Blue arrows indicate M1 polarized THP‐1 macrophages and red arrows indicate M2 polarized THP‐1 macrophages. THP‐1 macrophages were incubated with vehicle or LPS/IFNγ (100 ng/mL and 20 ng/mL, respectively, for 24 h or 48 h) with or without RIF (20 µm, for 48 h). K) qRT‐PCR analysis for M1 polarization‐related genes (*TNFα*, *NOS2*, and *CCL2*) and M2 polarization‐related genes (*TGM2*, *MRC1* and *CCL22*) in THP‐1 macrophages (n = 6). Data are shown as mean ± SD. ^*^ *p* < 0.05, ^**^ *p* < 0.01, and ^***^ *p* < 0.001 compared with the LPS/IFNγ group. *p* > 0.05 compared with the LPS/IFNγ group, ns: not significant.

The role of PXR in regulating polarization was further investigated in primary murine bone marrow‐derived macrophages (BMDMs), murine Raw264.7 and human THP‐1 macrophages. The in vivo experiments indicated that PXR activation significantly modulated the CD206/iNOS expression of murine hepatic macrophages. We further validated the regulatory effect in BMDMs by incubation with murine PXR agonist PCN. The BMDMs were isolated and differentiated with macrophage colony‐stimulating factor (M‐CSF) for 7 days (Figure S1C,D, Supporting Information). Immunofluorescence staining results showed that compared to BMDMs stimulated with LPS, PCN co‐incubation significantly increased the fluorescence intensity of CD206. Also, PXR activation in BMDMs markedly decreased the iNOS fluorescence intensity, which was consistent with the in vivo results (**Figure** **3**A–D).

NO is a pro‐inflammatory factor secreted by M1 macrophages, and M2 macrophages could reduce NO production, exerting an anti‐inflammatory effect. The effects of various concentrations of PCN on cell viability and NO production were first examined in Raw264.7 cells. PCN incubation reduced the NO levels induced by LPS stimulation in a concentration‐dependent manner (Figure S3A,B, Supporting Information). PXR in Raw264.7 cells was activated after incubation with 20 µm PCN for 48 h, as revealed by measuring the protein expression of the PXR target genes UGT1A1, CYP3A11 and GSTM2 (Figure S3D,E, Supporting Information). Moreover, LPS stimulation induced round and flattened cells with multiple pseudopodia (M1 polarization), while PCN induced elongated fusiform cells (M2 polarization) (Figure S3C, Supporting Information). As anticipated, PXR activation decreased the gene expression of M1 markers (*Tnfα*, *Nos2*, and *Il6*) and increased the expression of M2 markers (*Arg1*, *Mgl1*, *Fizz1*, and *Mrc2*) (Figure 3E; Figure S3F, Supporting Information). Additionally, to explore the polarization of Raw264.7 cells, CD86 and CD206 were selected as M1 and M2 markers, respectively. Flow cytometric analysis showed that PXR activation decreased the density of CD86^+^ cells from 62.2% to 42.3% and increased the percentage of CD206^+^ cells from 26.2% to 40.7% (Figure 3F,G). Immunofluorescence staining indicated that PCN markedly induced CD206 expression, with a 1.7‐fold increase in fluorescence intensity (Figure 3H,I).

The regulatory effect of PXR activation on polarization was explored in human THP‐1 macrophages. RIF at a concentration of 20 µm was incubated with THP‐1 macrophages (Figure S3G, Supporting Information). PXR in THP‐1 macrophages was successfully activated by incubation with 20 µm RIF for 48 h, as revealed by the upregulation of downstream proteins (Figure S3H,I, Supporting Information). Striking morphological alterations emerged in THP‐1 macrophages that were incubated with RIF. LPS and interferon‐γ (IFNγ) stimulated THP‐1 macrophages to polarize into the M1 phenotype with fusiform morphology, while the shape of cells that were incubated with RIF was polygonal (M2 polarization), and the morphological changes were more evident with prolonged incubation time (Figure 3J). Furthermore, compared with LPS/IFNγ group, RIF co‐incubation downregulated the elevated M1 markers, *TNFα* and C‐C motif chemokine ligand 2 (*CCL2*), and upregulated M2‐related markers, *TGM2*, *MRC1* and *CCL22* (Figure 3K). These results suggested that PXR activation could induce M1 to M2 macrophage transformation.

### 2.4 Depletion of Macrophages Abrogates the Hepatoprotective Effect of PXR Activation After LPS/GaIn Treatment in Mice

**Figure 4:**
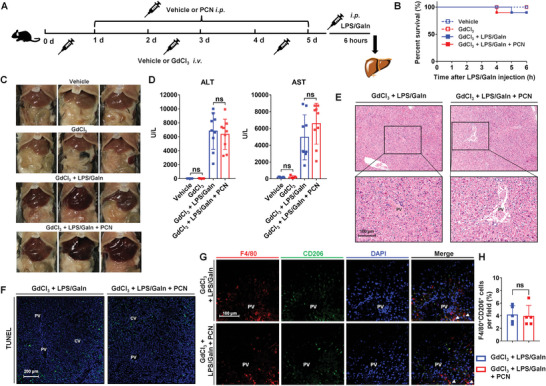
Macrophage depletion abrogates the protective effect of PXR activation. A) Mice were intravenously injected with GdCl_3_ (10 mg kg^−1^) every other day from Day 0, for a total of three times. PCN (100 mg kg^−1^/d) was intraperitoneally injected for 5 days beginning on Day 1, followed by an intraperitoneal injection of saline or LPS/GaIn (100 µg kg^−1^ and 700 mg kg^−1^, respectively) after the final injection of PCN (*n* = 5 for vehicle and GdCl_3_ groups, and *n* = 10 for GdCl_3_ + LPS/GaIn and GdCl_3_ + LPS/GaIn + PCN groups). B) Survival curves for 6 h after injection. C) Representative gross images of mouse livers. D) Serum ALT and AST levels. E,F) Representative H&E and TUNEL staining of the liver sections. G,H) Immunofluorescence staining and quantification of F4/80 (red) and CD206 (green). Filled triangles indicate F4/80^+^CD206^+^ cells (*n* = 5). The data are expressed as the mean ± SD. *p* > 0.05 compared with the vehicle or GdCl_3_ + LPS/GaIn groups, ns: not significant.

To confirm the contribution of macrophages to the protective effect of PXR activation on LPS/GaIn‐induced liver injury, mice were intravenously injected with gadolinium chloride (GdCl_3_) to deplete macrophages (**Figure** **4**A). GdCl_3_ injection reduced F4/80^+^ cells and *F4/80* mRNA expression in the liver (Figure S4A–C, Supporting Information). The depletion of macrophages did not influence the activation of PXR in the liver (Figure S4D,E, Supporting Information), but almost completely abrogated the protective effect against LPS/GaIn‐induced liver injury. After macrophage depletion, PXR activation did not improve the survival rates, and the LPS/GaIn and LPS/GaIn + PCN groups showed similarly elevated ALT and AST levels (Figure 4B,D). The livers in both LPS/GaIn‐treated groups showed severe congestion (Figure 4C). Pathological staining also revealed serious hepatic multifocal hemorrhages and extensive hepatocyte necrosis in the LPS/GaIn and LPS/GaIn + PCN groups after macrophage depletion (Figure 4E,F; Figure S4F, Supporting Information). The proportions of F4/80^+^CD206^+^ cells in the livers of these two groups were similarly infrequent (Figure 4G,H). These results indicated that macrophages performed important functions in PXR‐mediated protection against LPS/GaIn‐induced liver injury.

### 2.5 Loss of PXR in Macrophage Impairs the Hepatoprotective Effect of PXR Activation After LPS/GaIn Treatment in Mice

**Figure 5:**
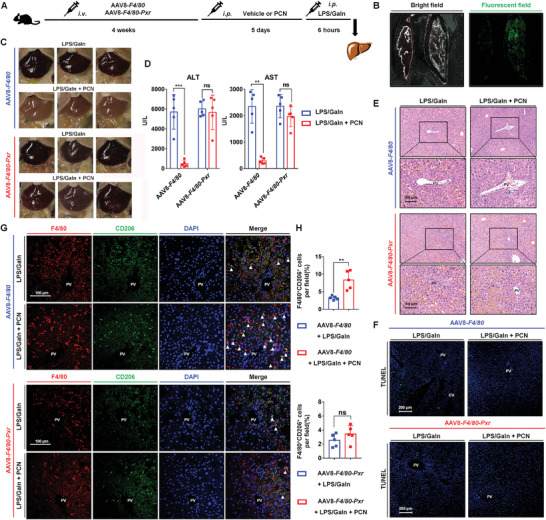
Ablation of PXR in macrophages impairs the protective effect of PXR activation against LPS/GaIn‐induced liver injury and the promotion of M2 polarization in macrophages. A) Mice were intravenously injected with AAV8‐*F4/80* or AAV8‐*F4/80*‐*Pxr* to generate macrophage‐specific *Pxr* deficiency models. After 4 weeks of infection, PCN (100 mg kg^−1^/d) was intraperitoneally injected for 5 days, and LPS/GaIn (100 µg kg^−1^ and 700 mg kg^−1^, respectively) was intraperitoneally injected. The mice were sacrificed 6 h after injection (*n* = 5). B) Representative fluorescent images of livers treated with saline (left tissue) and AAV8‐*F4/80*‐*Pxr* (right tissue). C) Representative gross images of mouse livers. D) Serum ALT and AST levels (*n* = 5). E,F) Representative H&E and TUNEL staining of liver sections. G,H) Double staining and quantification of F4/80 (red) and CD206 (green) (*n* = 5). Filled triangles indicate the F4/80^+^CD206^+^ cells. The data are expressed as the mean ± SD. ^**^ *p* < 0.01, ^***^ *p* < 0.001 compared with the AAV8‐*F4/80* + LPS/GaIn group, and *p* > 0.05 compared with the AAV8‐*F4/80*‐*Pxr* + LPS/GaIn group, ns: not significant.

To investigate whether PXR deficiency in macrophages affects the protection of PXR against LPS/GaIn‐induced liver injury, macrophage‐specific *Pxr*‐knockdown and negative control mice were generated by intravenously injecting with adeno‐associated virus 8 (AAV8)‐*F4/80*‐*Pxr*‐shRNA‐Zsgreen (AAV8‐*F4/80*‐*Pxr*) or AAV8‐*F4/80*‐shRNA‐Zsgreen (AAV8‐*F4/80*), respectively (**Figure** **5**A). Successful transfection was confirmed by the detection of Zsgreen fluorescence in the liver, and the knockdown of PXR in macrophages was reflected by double immunofluorescence staining (Figure 5B; Figure S5A,B, Supporting Information). The expression of PXR downstream proteins was detected and showed that selective knockdown of PXR in macrophages did not affect the activation of PCN treatment in the entire liver tissue (Figure S6A–D, Supporting Information). AAV8‐*F4/80*‐treated mice exposed to PCN had protection against LPS/GaIn‐induced liver injury. However, the AAV8‐*F4/80*‐*Pxr* group treated with PCN showed severe liver congestion and elevated serum ALT and AST levels (Figure 5C,D). Moreover, PCN treatment significantly ameliorated multifocal hemorrhages and hepatocyte necrosis in the livers of AAV8‐*F4/80*‐treated mice but failed to alleviate these effects in AAV8‐*F4/80*‐*Pxr*‐treated mice (Figure 5E,F; Figure S6E,F, Supporting Information). Compared with the LPS/GaIn‐treated mice, LPS/GaIn + PCN treatment also decreased the production of pro‐inflammatory cytokines IL6 and TNFα in the AAV8‐*F4/80* group. Whereas after *Pxr* knockdown in macrophages, PXR activation cannot decrease the secretion of TFNα and IL6 (Figure S6G,H, Supporting Information). Furthermore, the regulatory effect of PXR activation on macrophage polarization disappeared after treatment with AAV8‐*F4/80*‐*Pxr*. PXR activation did not reduce the number of F4/80^+^iNOS^+^ cells of mice in the AAV8‐*F4/80*‐*Pxr* group, while the number of F4/80^+^iNOS^+^ cells was significantly decreased in the AAV8‐*F4/80* group (Figure S6I,J, Supporting Information). Similarly, PXR activation increased the number of F4/80^+^CD206^+^ cells in mice in the AAV8‐*F4/80* group, but this effect was absent in macrophages lacking PXR (Figure 5G,H). Taken together, these results indicated that PXR in macrophages contributed to the protective and regulatory effects of PXR activation in the livers of LPS/GaIn‐treated mice.

### 2.6 PXR Activation Promotes Macrophage M2 Polarization through the STAT6 Pathway

**Figure 6:**
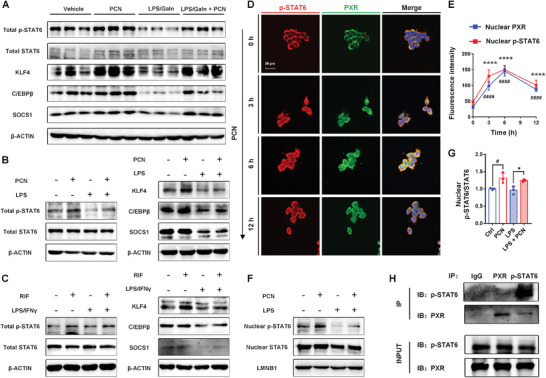
The effects of PXR activation on the STAT6 signaling pathway. A) The protein expression of total p‐STAT6, total STAT6, KLF4, C/EBPβ, and SOCS1 protein expression in mouse liver. B,C) The protein expression of total p‐STAT6, total STAT6, KLF4, C/EBPβ, and SOCS1 protein expression in B) Raw264.7 cells and C) THP‐1 macrophages. D) Confocal images of the nuclear translocation of PXR (green) and p‐STAT6 (red) in Raw264.7 macrophages at 0, 3, 6, and 12 h after PCN incubation. E) Quantification of the fluorescence intensity of nuclear PXR and nuclear p‐STAT6 in Raw264.7 cells (*n* = 5). The data are expressed as the mean ± SD. ^####^ *p* < 0.0001 compared with nuclear PXR at 0 h. ^****^ *p* < 0.0001 compared with the nuclear p‐STAT6 at 0 h. F,G) Western blotting and quantification of nuclear p‐STAT6 and nuclear STAT6 protein expression in Raw264.7 cells (*n* = 3). The data are expressed as the mean ± SD. ^#^ *p* < 0.05 compared with the control group. ^*^ *p* < 0.05 compared with the LPS group. H) Co‐IP analysis of PXR and p‐STAT6 in primary Kupffer cells.

STAT6 is considered a key regulator of cell polarization and differentiation; thus, the expression of related proteins in the STAT6 pathway was measured. The p‐STAT6/STAT6 ratio was significantly increased in the LPS/GaIn + PCN group compared to the LPS/GaIn group in mice. Similarly, the expressions of STAT6 downstream proteins and M2 polarization‐associated proteins, including KLF4, C/EBPβ and SOCS1, were increased by PXR activation (**Figure** **6**A; Figure S7A, Supporting Information). To examine whether PXR modulated the STAT6 pathway in vitro, murine Raw264.7 cells and human THP‐1 macrophages were used. PXR activation also increased the p‐STAT6/STAT6 ratio, and the protein expressions of KLF4, C/EBPβ and SOCS1 in both Raw264.7 cells incubated with PCN (Figure 6B; Figure S7B, Supporting Information) and THP‐1 macrophages incubated with RIF (Figure 6C; Figure S7C, Supporting Information).

PXR and p‐STAT6 are commonly located in the cytoplasm and translocate into the nucleus after activation.^[^ ^14^, ^17^ ^]^ To investigate the interaction of PXR and p‐STAT6, co‐localization and coimmunoprecipitation (co‐IP) analyses were conducted. Activation of PXR by PCN enhanced the translocation of p‐STAT6 to the nucleus in Raw264.7 cells. The changes in the nuclear fluorescence intensities of p‐STAT6 and PXR were almost synchronized, increasing gradually from 0–6 h and decreasing after 6 h (Figure 6D,E). The protein expressions of nuclear p‐STAT6 and STAT6 were also measured, as the result of a significant increase in the p‐STAT6/STAT6 ratio in the nuclei with PCN treatment (Figure 6F,G). Additionally, endogenous PXR selectively bound to p‐STAT6 in primary Kupffer cells (Figure 6H).

**Figure 7:**
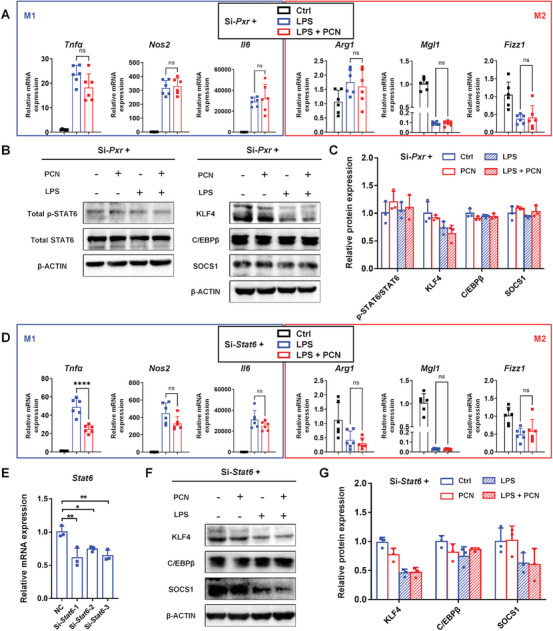
PXR and STAT6 regulate macrophage M2 polarization. Raw264.7 cells were treated with siRNA for 24 h to silence *Pxr* prior to incubation with vehicle or LPS (1 µg mL^−1^, for 24 h) with or without PCN (20 µM, for 48 h). A) qRT‐PCR analysis of M1 polarization‐related genes (*Tnfα*, *Nos2*, and *Il6*) and M2 polarization‐related genes (*Arg1*, *Mgl1*, and *Fizz1*) in Raw264.7 cells after treatment with si‐*Pxr* (*n* = 6). B, C) Western blotting and quantification of total p‐STAT6, total STAT6, KLF4, C/EBPβ, and SOCS1 protein expressions in Raw264.7 cells after si‐*Pxr* treatment (*n* = 3). The data are expressed as the mean ± SD. *p* > 0.05 compared with the LPS group, ns: not significant. Raw264.7 cells were treated with siRNA for 24 h to silence *Stat6* prior to incubation with vehicle or LPS (1 µg mL^−1^, for 24 h) with or without PCN (20 µm, for 48 h). D) qRT‐PCR analysis of M1 polarization‐related genes (*Tnfα*, *Nos2*, and *Il6*) and M2 polarization‐related genes (*Arg1*, *Mgl1*, and *Fizz1*) in Raw264.7 cells after si‐*Stat6* treatment (*n* = 6). The data are expressed as the mean ± SD. ^****^ *p* < 0.0001 compared with the LPS group. *p* > 0.05 compared with the LPS group, ns: not significant. E) qRT‐PCR analysis of the interference efficiency of si‐*Stat6* toward *Stat6* mRNA levels (*n* = 3). The data are expressed as the mean ± SD. ^*^ *P* < 0.05 and ^**^ *P* < 0.01 compared with the negative control (NC) group. F,G) Western blotting and quantification of KLF4, C/EBPβ, and SOCS1 protein expressions in Raw264.7 cells after si‐*Stat6* treatment (*n* = 3).

To assess whether PXR‐mediated promotion of M2 polarization was dependent on STAT6, siRNAs were used to silence *Pxr* (Figure S7D,E and Table S1, Supporting Information) and *Stat6* in Raw264.7 cells. After *Pxr* silencing, the expression of PXR target proteins remained unchanged after activation (Figure S7F,G, Supporting Information). Silencing *Pxr* in Raw264.7 cells completely abrogated the upregulation of M2 gene expression (*Arg1*, *Mgl1*, *Fizz1*, and *Mrc2*) and the downregulation of M1 gene expression (*Tnfα*, *Nos2*, and *Il6*) (**Figure** **7**A; Figure S7H, Supporting Information). PXR activation after si‐*Pxr* treatment failed to modulate the STAT6 pathway and M2 polarization‐associated proteins (p‐STAT6, KLF4, C/EBPβ, and SOCS1) (Figure 7B,C). Additionally, Raw264.7 cells were treated with si‐*Stat6* to identify the contribution of the STAT6 pathway. Si‐*Stat6*‐1 was examined by using silencing efficiency screening (Figure 7E; Table S1, Supporting Information). *Stat6* silencing did not influence the activation of PXR in Raw264.7 cells, as shown by the increased protein expression of downstream genes after PXR activation (Figure S7I,J, Supporting Information). However, after *Stat6* silencing, the effect of PXR activation on the polarization (M1 markers: *Nos2* and *Il6*, M2 markers: *Arg1*, *Mgl1*, and *Fizz1*) and regulation of the STAT6 pathway (KLF4, C/EBPβ, and SOCS1) disappeared (Figure 7D,F,G). Overall, the activation of PXR promoted macrophage M2 polarization by interacting with p‐STAT6 and promoting nuclear translocation.

## 3 Discussion

Liver damage is the major complication of endotoxemia and sepsis and has high relevance to mortality.^[^ ^2^ ^]^ The balance between pro‐inflammatory and anti‐inflammatory macrophages serves to maintain homeostasis and defense, and a shift toward the M2 phenotype may protect against inflammatory diseases and promote adverse tissue regeneration following liver injury. Therefore, various treatments based on shifting macrophage polarization have been explored.^[^ ^18^ ^]^ The current study revealed that PXR activation played a protective role in LPS/GaIn‐induced liver injury through a macrophage‐related mechanism.

The tissue distribution of PXR parallels its function, with substantial expression in the liver. Our initial observation of PXR expression in macrophages/Kupffer cells prompted us to further explore its role in the immune response. Macrophages are immune cells that contribute to pathogenic microorganism defense and tissue repair, and polarization is regarded as an essential feature of functional switching.^[^ ^19^ ^]^ In endotoxin‐induced liver injury, macrophages are activated by LPS and polarize to the M1 phenotype. By generating pro‐inflammatory mediators such as TNFα, IL6, and NO, M1 cells result in other immunocytes infiltration, vasodilation, and hemodynamic abnormalities in the liver, leading to congestion, edema, and necrosis of liver tissue.^[^ ^20^ ^]^ In contrast, M2 cells secrete anti‐inflammatory cytokines, such as IL10 and TGFβ, suppressing the inflammatory response and counteracting the extension of inflammation.^[^ ^20^ ^]^ Current results identify PXR as a novel regulator of macrophage polarization. Our in vitro study provided evidence showing that PCN induced the transformation of M1 to M2 in macrophages in response to LPS stimulation. Indeed, once PXR was specifically silenced in macrophages, the effect of PXR activation on promoting M2 polarization was absent, and the resulting protective effect against endotoxin‐induced liver injury was lost.

STAT6 is a key transcription factor in macrophage M2 polarization, which is closely linked to immune and inflammatory processes. When macrophages are stimulated by certain cytokines, such as IL4 and IL13, STAT6 is phosphorylated and enters the nucleus to regulate the transcription of genes associated with M2 polarization.^[^ ^21^ ^]^ Our study showed that the PXR‐induced upregulation of M2 markers was abolished after STAT6 knockdown using si‐*Stat6*, indicating that STAT6 is essential for PXR‐induced M2 polarization. Moreover, STAT6 has also been demonstrated to suppress M1 polarization by inhibiting the nuclear factor‐kappaB (NF‐κB) signaling pathway through multiple mechanisms, including inhibiting the degradation of inhibitor of kappa B alpha, competing with NF‐κB for binding to the required coactivators and upregulating anti‐inflammatory cytokines.^[^ ^22^ ^]^ Our study showed that PXR activation downregulated the M1 marker TNFα, which is also a classical target gene of NF‐κB. This downregulation was absent after *Pxr* silencing, while significant inhibition still existed after the knockdown of *Stat6*. These results suggested that PXR may inhibit TNFα via the NF‐κB pathway, which was also consistent with previous studies.^[^ ^23^ ^]^ Additionally, the C‐terminal transcriptional activation domain in the STAT6 structure is responsible for nuclear translocation and recruitment of other proteins and coactivators to facilitate the transcription of target genes.^[^ ^14^ ^]^ Nuclear receptor coactivator 1 (NCOA1) is a coactivator required for the transcriptional activity of STAT6 that promotes the transcriptional regulatory process of STAT6 by binding directly to the transcriptional activation domain.^[^ ^18^, ^24^ ^]^ Previous studies have reported that PXR can recruit NCOA1 to the activation function‐2 region of the PXR ligand binding domain.^[^ ^25^ ^]^ The protein‐protein interaction between PXR and p‐STAT6 was also examined in our study and showed that PXR selectively bound to p‐STAT6 in primary Kupffer cells. However, whether NCOA1 is involved in the protein‐protein interaction between PXR and p‐STAT6 remains unclear, and further studies are needed to clarify their relationship.

Although PXR is largely known for regulating drug metabolism, recent studies have explored the role of PXR in regulating immune responses to challenges with xenobiotic exposure.^[^ ^26^ ^]^ It was reported that PXR‐deficient mice were vulnerable to LPS/GaIn‐induced liver injury.^[^ ^27^ ^]^ Previous studies showed that activating PXR inhibited the proliferation and activation of human T lymphocytes, and the development of B lymphocytes in the fetal liver.^[^ ^11^, ^26^ ^]^ Moreover, clinical and laboratory data showed that RIF, a clinical antituberculosis drug, exerted potent immunosuppressive effects.^[^ ^13^, ^28^ ^]^ RIF inhibited the secretion of pro‐inflammatory cytokines in purified human peripheral blood monocytes, such as TNFα.^[^ ^29^ ^]^ Consistent with this, we observed that RIF promoted M2 polarization and inhibited M1 polarization in hMDMs and THP‐1 macrophages. The immunomodulatory effects of PXR are receiving increasing attention and are gradually being translated into clinical practice. A recent retrospective study showed that rifaximin reduced the incidence of sepsis in patients with cirrhosis awaiting liver transplantation.^[^ ^30^ ^]^ Furthermore, rifaximin administration was shown to exert a protective effect against early liver injury in liver transplant patients, possibly by suppressing inflammatory cell activation.^[^ ^31^ ^]^

In conclusion, we have demonstrated that PXR activation protected against endotoxemic liver injury by inhibiting M1 polarization and promoting M2 polarization of macrophages. Therefore, PXR agonists showed a regulatory effect on macrophage polarization, which may represent a novel treatment strategy for endotoxemia‐related liver injury.

## 4 Experimental Section

### hMDM

Twelve healthy volunteers (seven men and five women, ethical approval number: SYSKY‐2023‐874‐01) were enrolled in the studies. Monocytes were centrifuged and collected from healthy volunteers using an extraction kit (Solarbio, Beijing, China) and differentiated with 100 ng mL^−1^ granulocyte‐macrophage colony‐stimulating factor (GM‐CSF, Abclonal, Wuhan, China) for 7 days.^[^ ^32^ ^]^ The differentiation of hMDM was confirmed by qRT‐PCR and flow cytometry. hMDMs were cultured in RPMI 1640 medium (Invitrogen, Carlsbad, USA) supplemented with 10% FBS (ZETA Life, CA, USA), and 1% penicillin‐streptomycin at 37 °C with 5% CO_2_. After differentiation, hMDMs were incubated with RIF (20 µM, Sigma, St. Louis, USA) or DMSO for an additional 48 h. Experiments were performed in mycoplasma‐free cells.

### Animal Experiments

Male C57BL/6J mice aged 8–10 weeks were obtained from the Guangdong Medical Laboratory Animal Center (Foshan, China). *PXR*‐humanized mice were purchased from PharmaSS corporation (Zhongshan, China). The mice were housed in conditions with standard temperature and humidity control and a 12 h light/dark cycle (ethical approval number: SYSU‐IACUC‐2019‐B212).

*Mouse Treatments*: Eight‐ to 10‐week‐old male mice were intraperitoneally injected with 100 mg kg^−1^/d PCN (Sigma, St. Louis, USA) or vehicle (corn oil, Sigma, St. Louis, USA) for 5 days. Two hours after the last PCN injection, saline or LPS/GaIn (100 µg kg^−1^ and 700 mg kg^−1^, respectively. Sigma, St. Louis, USA) was administered intraperitoneally. Liver tissues and blood samples were collected 6 h after LPS/GaIn injection.

*Macrophage Depletion*: As previously described,^[^ ^33^ ^]^ 10 mg kg^−1^ GdCl_3_ (TargetMol, Boston, USA.) was intravenously injected into mice every 2 days and for a total of 3 times to deplete macrophages. PCN (100 mg kg^−1^/d) was intraperitoneally injected for 5 days beginning on the day after the GdCl_3_ injection. Two hours after the last PCN administration, LPS /GaIn (100 µg kg^−1^ and 700 mg kg^−1^, respectively) was intraperitoneally injected, and liver tissues and blood samples were collected 6 h after LPS/GaIn injection.

*Macrophage‐Specific PXR Interfere*: Macrophage‐specific *Pxr* interference mice were generated using AAV8 viral vectors that delivered a macrophage‐specific promoter (*F4/80*), encoded *Pxr* shRNA and co‐expressed the ZsGreen reporter gene. Macrophage‐specific *Pxr*‐knockdown and negative control mice were generated by intravenous injection of AAV8‐*F4/80*‐*Pxr* or AAV8‐*F4/80* (1.0 × 10^12^ genome copies per mouse, Hanbio, Shanghai, China). After 4 weeks of viral infection, PCN (100 mg kg^−1^/d) was intraperitoneally injected for 5 consecutive days to activate hepatic PXR. LPS/GaIn (100 µg kg^−1^ and 700 mg kg^−1^, respectively) was intraperitoneally injected 2 h after the last PCN administration, and 6 h later, blood samples and liver tissues were collected.

### Primary Kupffer Cell Isolation

Primary Kupffer cells were isolated from C57BL/6J mice (8–10 weeks) and separated by gradient centrifugation according to the literature.^[^ ^34^ ^]^ The liver was perfused with calcium‐ and magnesium‐free HBSS containing 0.5 mM EGTA and digested with 0.075% type IV collagenase (Yuanye Bio‐Technology, Shanghai, China) in DMEM. The liver was then transferred, and the capsule was peeled off. After being repeatedly homogenized in DMEM (Corning, New York, USA), the liver was further digested in an incubator shaker at a speed of 80 rpm at 37 °C for 20 min. The cell suspensions were filtered through a sterile nylon mesh (150 µm) and subsequently centrifuged at 50 × g for 5 min three times to remove hepatocytes. The supernatant was then centrifuged at 500 × g for 15 min to precipitate nonparenchymal cells. The cell pellets were resuspended in Percoll working solution and then centrifuged at 800 × g at room temperature for 15 min. Kupffer cells were collected and washed with DMEM.

### BMDM

BMDMs were isolated and differentiated according to standard protocols.^[^ ^35^ ^]^ Briefly, mice were euthanized by rapid cervical dislocation, the femur and tibia were isolated from the mouse leg. The bones proximal to each joint were cut and rinsed with BMDM differentiation buffer to collect mouse bone marrow cells. Cell suspension was filtered through 70 µm and centrifuged at 500 × g for 3 min at 4 °C. BMDMs were differentiated with M‐CSF (Meilunbio, Dalian, China) at 25 ng mL^−1^ for 7 days. To confirm the successful differentiation of BMDMs, the F4/80 mRNA and surface protein levels were determined before and after induction using qRT‐PCR and flow cytometry, respectively. BMDMs were cultured in DMEM with 20% FBS and 1% penicillin‐streptomycin at 37 °C with 5% CO_2_. After differentiation, BMDMs were incubated with LPS (1 µg mL^−1^, 6 h), and with or without PCN (20 µm, 48 h). Experiments were performed in mycoplasma‐free cells.

### Cell Culture and Treatment Protocol

Murine Raw264.7 macrophages were purchased from Cellcook (Guangzhou, China) and cultured in DMEM with 12% FBS and 1% penicillin‐streptomycin at 37 °C with 5% CO_2_. To investigate the effect of PXR activation on polarization, Raw264.7 cells were preincubated with 20 µm PCN or DMSO for 24 h. After preincubation, the cells were incubated with 1 µg mL^−1^ LPS with PCN or DMSO for an additional 24 h.

Human THP‐1 macrophages were purchased from Procell (Wuhan, China) and cultured in RPMI 1640 medium supplemented with 10% FBS, 0.05 mm β‐mercaptoethanol (Procell, Wuhan, China), and 1% penicillin‐streptomycin at 37 °C with 5% CO_2_. To differentiate THP‐1 cells into macrophages, the cells were incubated with 320 nm PMA for 24 h. THP‐1 macrophages were stimulated by LPS (100 ng mL^−1^) and human IFN‐γ (20 ng mL^−1^) for 24 h to induce M1 polarization.^[^ ^36^ ^]^ To explore the effect of PXR activation on THP‐1 macrophage polarization, the cells were preincubated with 20 µm RIF or DMSO before being polarized into the M1 phenotype. After preincubation, THP‐1 macrophages were incubated with LPS (100 ng mL^−1^) and IFN‐γ (20 ng mL^−1^). PMA and IFN‐γ were purchased from Beyotime (Shanghai, China).

### Histopathological Analysis and TUNEL Assay

Liver tissues were embedded in paraffin and sectioned at 4 µm. The sections were then stained with H&E (Servicebio, Wuhan, China) and imaged using a NIKON ECLIPSE CI microscope (Nikon Instruments, Japan). To detect cell death in the liver, sections were stained for nuclear DNA fragmentation with TUNEL (Beyotime, Shanghai, China). Cell nuclei were counterstained with DAPI (Beyotime, Shanghai, China). Images were captured using FV3000 confocal microscope (Olympus, Tokyo, Japan).

### ALT and AST Measurement

Serum samples were obtained by centrifugation at 3500 rpm for 15 min. ALT and AST levels were measured by URIT 8021A automated biochemical analyzer from URIT Medical Electronic Co., Ltd (Guilin, China).

### Immunofluorescence and Immunohistochemistry Staining

Liver paraffin sections were deparaffinized and hydrated, followed by antigen retrieval by heating in citric acid buffer (pH 6.0) for 30 min. Sections were blocked with goat or donkey serum or BSA for 1–2 h at room temperature. Sections were incubated with primary antibodies at 4 °C overnight, followed by incubation with secondary antibodies at room temperature for 1–2 h. Cell nuclei were counterstained with DAPI for immunofluorescence staining. Cells were counterstained with hematoxylin for immunohistochemistry staining. Stained sections were photographed with an FV3000 confocal microscope (Olympus, Tokyo, Japan).

The rabbit anti‐F4/80 and anti‐ARG1 antibodies were purchased from Cell Signaling Technology (Danvers, USA), the rat anti‐CLEC4F and the goat anti‐CD206 antibodies were purchased from R&D Systems (Minneapolis, USA), the mouse anti‐PXR, anti‐IL6 and anti‐CD206 antibodies were purchased from Santa Cruz Biotechnology (CA, USA) and the rabbit anti‐PXR and anti‐IL10 antibodies were obtained from ABclonal (Wuhan, China). The mouse anti‐TNFα antibody was purchased from Proteintech Group (Wuhan, China). The rabbit anti‐CD68 antibody was obtained from Abcam (Cambridge, UK). The rat anti‐F4/80 antibody was purchased from eBioscience (Thermo Fisher Scientific, MA, USA). The anti‐mouse IgG Alexa Fluor 488 and anti‐rabbit IgG Alexa Fluor 647 secondary antibodies were obtained from Cell Signaling Technology (Danvers, USA), and the anti‐goat IgG Alexa Fluor 555 and anti‐rat IgG Alexa Fluor 488 antibodies were purchased from Abcam (Cambridge, UK).

### Co‐IP

Primary Kupffer cells from male C57BL/6J mice (8–10 weeks) were isolated according to the method described. The co‐IP experiment was performed using the Thermo Scientific Pierce co‐IP kit (Thermo Scientific, Rockford, USA) according to the manufacturer's introduction. The co‐IP samples were analyzed by western blotting using anti‐PXR (Santa Cruz Biotechnology, CA, USA) and anti‐p‐STAT6 (Cell Signaling Technology, Danvers, USA). Anti‐rabbit or anti‐mouse IgG was purchased from Abclonal, Wuhan, China).

### Cell Viability and NO Content Detection

Raw264.7 cells were seeded in a 96‐well plate at a density of 5 × 10^4^ cells/mL, and THP‐1 cells were seeded at a density of 10 × 10^4^ cells/mL. After 48 h of incubation, 100 µL serum‐free medium containing 10 µL CCK‐8 solution was added per well. Cells were incubated for 2 h at 37 °C, and absorbance was read at 450 nm using a microplate reader (Thermo Scientific, Rockford, USA).

The NO content in the cell supernatant was measured using the NO content assay kit (Nanjing Jiancheng Bioengineering Institute, Nanjing, China) according to the manufacturer's instructions.

### Flow Cytometry Analysis

Single‐cell suspensions (1 × 10^7^ cells/mL) were prepared in PBS using a 70 µm nylon cell strainer. To block nonspecific binding, Raw264.7 cells were incubated with the mouse anti‐FcR antibody CD16/32 (Elabscience, Wuhan, China) for 10 min at room temperature. The cells were stained with anti‐CD86‐PE (Elabscience, Wuhan, China) at 4 °C for 30 min. The cells were fixed at room temperature for 1 h and incubated with anti‐CD206‐APC (Elabscience, Wuhan, China) in the permeabilization solution (Elabscience, Wuhan, China) for 30 min.^[^ ^37^ ^]^ hMDMs were blocked with the human Fc receptor blocking solution (BioLegend, CA, USA) and stained with anti‐CD14‐FITC (4A Biotech, Suzhou, China), anti‐CD163‐Percp/Cy5.5 or anti‐CD80‐PE (Elabscience, Wuhan, China). Cells were measured using flow cytometry and analyzed by FlowJo software (Tree Star Inc, OR, USA).

### Co‐Localization

Raw264.7 macrophages were incubated with 20 µm PCN for 0, 3, 6 and 12 h, and then fixed in the fixative solution (Beyotime, Shanghai, China) for 30 min. The cells were then permeabilized with 0.1% Triton X‐100 solution for 10 min. After permeabilization, the cells were incubated with primary antibodies for PXR (Santa Cruz Biotechnology, CA, USA) and p‐STAT6 (Cell Signaling Technology, Danvers, USA) at 4 °C overnight. After washing three times with PBS, the cells were incubated with the fluorescent secondary antibodies (anti‐mouse IgG Alexa Fluor 488 and anti‐rabbit IgG Alexa Fluor 647, Cell Signaling Technology, Danvers, USA) for 1 h at room temperature. DAPI was used to counterstain the cellular nuclei. Images were acquired by using an FV3000 confocal microscope (Olympus, Tokyo, Japan).

### Transfection of SiRNA

Silencing of *Pxr* or *Stat6* in Raw264.7 cells was performed using siRNA (Ribobio, Guangzhou, China). Raw264.7 macrophages were seeded into six‐well plates. Transfection conditions were according to the manufacturer's instructions. After siRNA interference for 24 h, cells were incubated with PCN (20 µM, 48 h) with or without LPS (1 µg mL^−1^, 24 h). Sequences of siRNAs are listed in Table S1 (Supporting Information).

### qRT‐PCR Analysis

Total RNA was isolated from Kupffer cells, Raw264.7 macrophages, or THP‐1 macrophages using Trizol reagent (Invitrogen, New York, USA), as described previously.^[^ ^38^ ^]^ After quantitative analysis by NanoDrop spectrophotometer (Thermo Scientific, Rockford, USA), 1 µg of total RNA was reverse transcribed into complementary DNA (cDNA) after the removal of DNA contamination using a PrimeScript RT reagent kit (Accurate Biology, Changsha, China). Amplification of cDNA was performed using the SYBR Green pro‐Taq HS qPCR kit (Accurate Biology, Changsha, China) in the ABI‐Prism 7500 Sequence Detection System (Applied Biosystems, Foster City, USA). All data were normalized to the housekeeping gene. Primers are listed in Tables S2 and S3 (Supporting Information).

### Western Blotting Analysis

Western blotting was performed as described in the previous report.^[^ ^17^ ^]^ Protein samples of liver tissue or cells were extracted using RIPA lysis buffer containing PMSF and phosphatase inhibitors (Biocolors, Shanghai, China). The nuclear proteins of Raw264.7 and THP‐1 macrophages were prepared using a nuclear/cytosol extract kit (Sangon Tech, Shanghai, China). The proteins were separated by electrophoresis on polyacrylamide gel (8–15% SDS gel) and then transferred to polyvinylidene fluoride membranes (0.2 or 0.45 µm, Millipore, Bedford, USA). After blocking in nonfat milk or BSA at room temperature for 1–2 h, the membranes were incubated with the primary antibody overnight at 4 °C. The membranes were then incubated with the secondary antibody for 2 h at room temperature. Chemiluminescence was detected using the electrochemiluminescence detection kit (Millipore, Bedford, USA) and captured using Tanon 5200 auto imaging system (Shanghai, China).

Mouse monoclonal anti‐PXR antibody was purchased from Proteintech Group (Wuhan, China). Rabbit polyclonal anti‐MDR1 antibody was purchased from Abcam (Cambridge, UK). Mouse monoclonal anti‐CYP3A was purchased from Santa Cruz Biotechnology (CA, USA). Rabbit anti‐KLF4, rabbit polyclonal anti‐GAPDH, rabbit monoclonal anti‐STAT6, anti‐p‐STAT6, anti‐SOCS1, and anti‐β‐ACTIN antibodies were purchased from Cell Signaling Technology (Danvers, USA). Mouse monoclonal anti‐LMNB1, and rabbit polyclonal anti‐GSTM2, rabbit polyclonal anti‐C/EBPβ, anti‐rabbit or anti‐mouse immunoglobulin G antibodies were purchased from Sangon Biotechnology (Shanghai, China). Rabbit polyclonal anti‐UGT1A1 antibody was acquired from Beyotime Biotechnology (Shanghai, China). Dilution rates of all antibodies were as indicated.

### Statistics

Paired data were analyzed using a paired t‐test. Unpaired Student's t‐test or multiple t‐tests was used for statistical analysis of unpaired data. Differences across multiple groups with one variable were compared using a one‐way analysis of variance (ANOVA) using GraphPad Prism 5 (GraphPad Software Inc, San Diego, USA). *P* values <0.05 were considered significant. The TOC figure was created using Figdraw.

### Ethic Approval Statement

Ethical approval to conduct the study with human blood samples was obtained from the Sun Yat‐Sen Memorial Hospital (ethical approval number: SYSKY‐2023‐874‐01), and all experiments were conducted according to the national and institutional guidelines. Healthy donors provided written informed consent prior to their participation in the study. All animal experiments were performed in accordance with the ARRIVE guidelines and approved by the Institutional Animal Care and Use Committee of Sun Yat‐Sen University (ethical approval number: SYSU‐IACUC‐2019‐B212).

## Conflict of Interest

The authors declare no conflict of interest.

## Author Contributions

T.Z. and G.Z. contributed equally to this work. Y.J., H.B., and M.H. conceptualized the project. Y.J. and T.Z. designed experiments. T.Z. and G.Z. performed basic experiments. R.C., S.S., and Y.L. provided the method supports. Y.W., G.W., and H.S. provided valuable intellectual input throughout. T.Z., Y.J., and H.B. drafted the manuscript. Y.J., H.B., and M.H. supervised the study.

## Supporting information

Supporting Information

## Data Availability

The data that support the findings of this study are available from the corresponding author upon reasonable request.
