# Supplementary material for: Pregnane X Receptor Activation in Liver Macrophages Protects against Endotoxin‐Induced Liver Injury
Source: Adv Sci (Weinh). 2024 Mar 13;11(19):2308771. doi: 10.1002/advs.202308771 (PMC11109625; doi:10.1002/advs.202308771)
Supplement: Supplementary file 1 — Supporting Information [file ADVS-11-2308771-s001.pdf]

ADVANCED SCIENCE
Open Access

Supporting Information

for *Adv. Sci.*, DOI 10.1002/advs.202308771

Pregnane X Receptor Activation in Liver Macrophages Protects against Endotoxin-Induced Liver Injury

Tingting Zhao, Guoping Zhong, Ying Wang, Renjie Cao, Shaofei Song, Yuan Li, Guohui Wan, Haiyan Sun, Min Huang, Huichang Bi* and Yiming Jiang**

Supporting information

Pregnane X receptor activation in liver macrophages protects against endotoxin-induced liver injury

Tingting Zhao, Guoping Zhong, Ying Wang, Renjie Cao, Shaofei Song, Yuan Li, Guohui Wan, Haiyan Sun, Min Huang, Huichang Bi, Yiming Jiang

1. Supplemental Figures

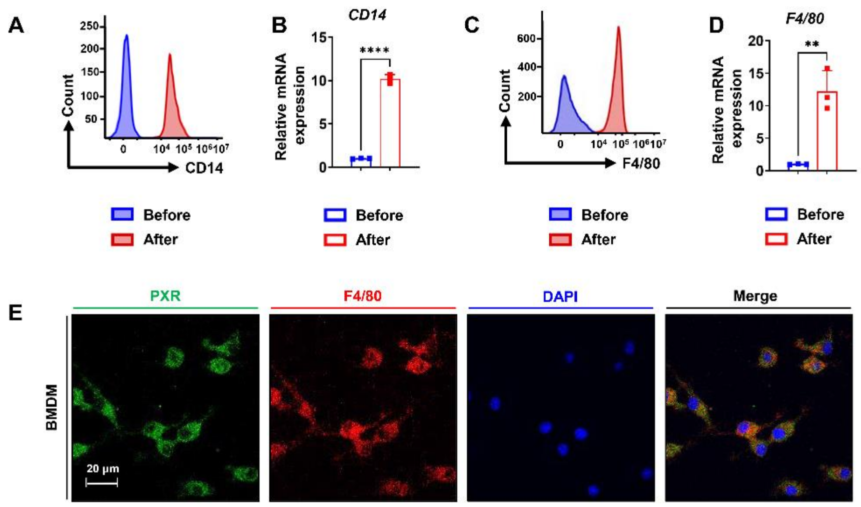

Figure S1. Detection of macrophage markers and PXR expression. A) Histogram of CD14$^+$ cells before and after differentiation (100 ng/mL GM-CSF for 7 days) of hMDMs. B) The mRNA level of *CD14* in hMDMs before and after differentiation (n = 3). C) Histogram of F4/80$^+$ cells before and after differentiation (25 ng/mL M-CSF for 7 days) of BMDMs. D) The mRNA level of *F4/80* in BMDMs before and after differentiation (n = 3). E) Immunofluorescence staining of BMDMs with PXR (green) and F4/80 (red). The data are expressed as the mean ± SD. $^{**}P < 0.01$ and $^{****}P < 0.0001$ as hMDMs or BMDMs after differentiation compared with before.

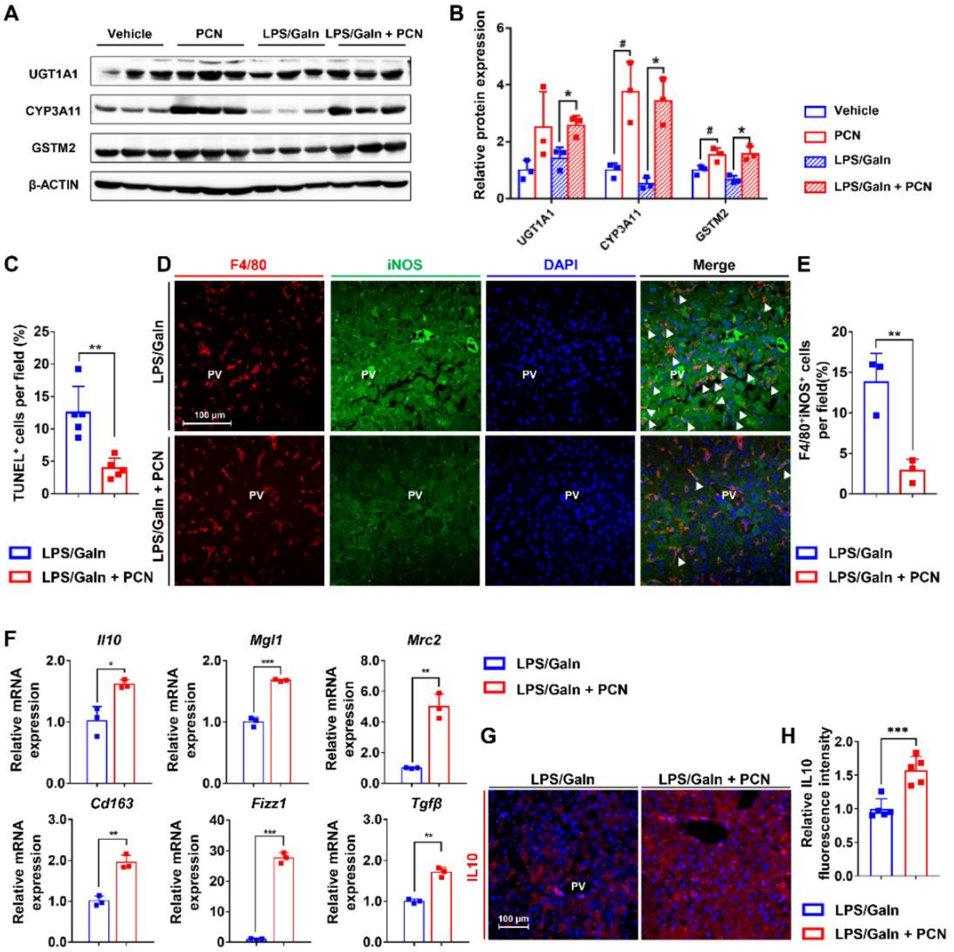

Figure S2. PXR activation protects against LPS/GaIn-induced liver injury and promotes macrophage M2 polarization. A, B) Western blotting and quantification of the protein expression of PXR downstream proteins in the liver (n = 3). C) Quantification of TUNEL$^+$ cells in liver sections (n = 5). D, E) Immunofluorescence staining and quantification of F4/80 (red) and iNOS (green) in LPS/GaIn and LPS/GaIn + PCN groups (n = 3). Filled triangles indicate the F4/80$^+$iNOS$^+$ cells. F) qRT-PCR analysis of M2 polarization-related genes (*Il10*, *Mgl1*, *Mrc2*, *Cd163*, *Fizz1* and *Tgfβ*) in primary Kupffer cells isolated from mice treated with LPS/GaIn and LPS/GaIn + PCN (n = 3). G, H) Immunofluorescence staining and quantification of IL10 in LPS/GaIn and LPS/GaIn + PCN groups (n = 5). Data are expressed as the mean ± SD. $^{\#}P < 0.05$ compared with the vehicle group. $^{*}P < 0.05$, $^{**}P < 0.01$ and $^{***}P < 0.001$ compared with the LPS/GaIn group.

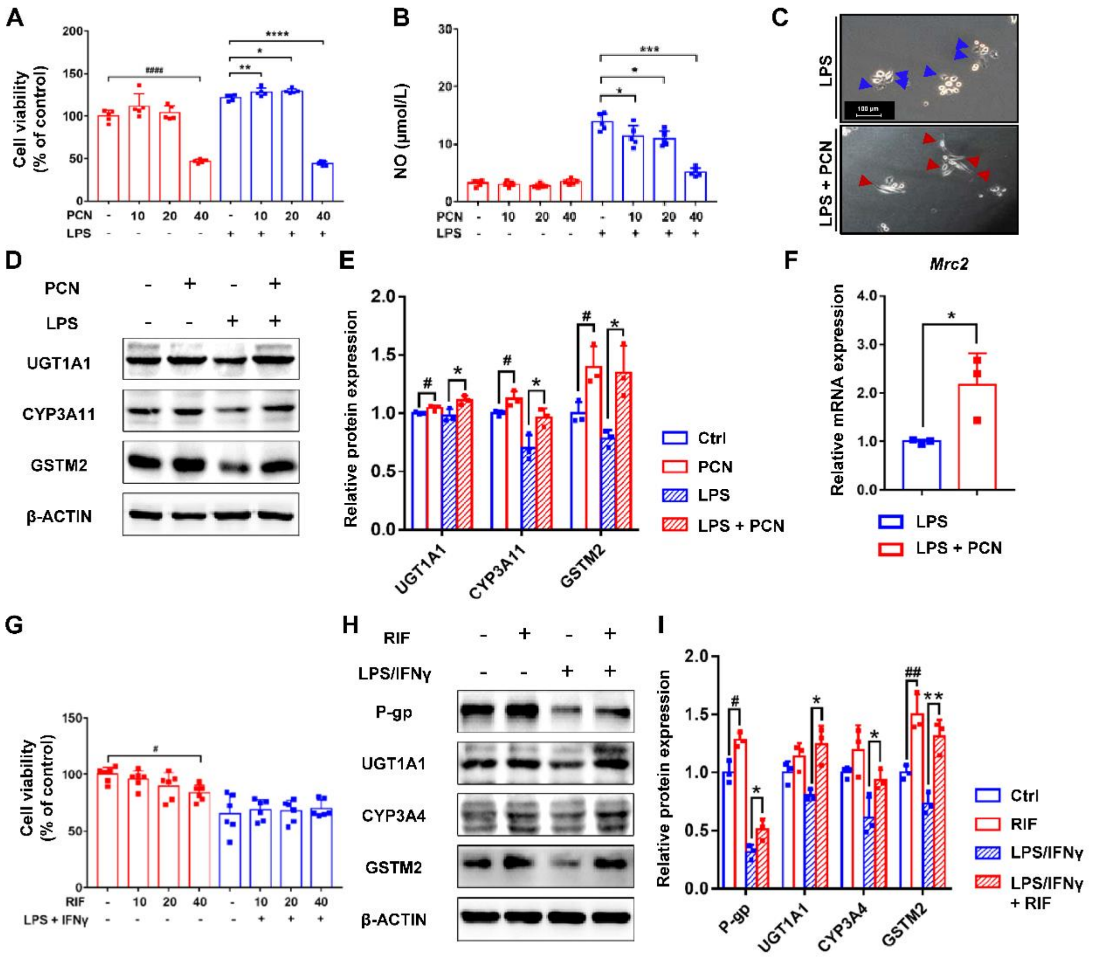

Figure S3. The effects of PXR activation on macrophages *in vitro*. A) The viability of Raw264.7 cells after incubation with various concentrations of PCN (0-40 μM, for 48 h) with or without LPS (1 μg/mL, for 24 h) (n = 5). B) NO levels in the supernatants of Raw264.7 cells incubated with PCN (0-40 μM, for 48 h) with or without LPS (1 μg/mL, for 24 h) (n = 5). C-F) Raw264.7 cells were incubated with LPS (1 μg/mL, for 24 h) with or without PCN (20 μM, for 48 h). C) Cell morphological changes of Raw264.7 cells after incubation with LPS or LPS + PCN. Blue arrows indicate M1 polarization and red arrows indicate M2 polarization. D, E) Western blotting and quantification of the protein expression of PXR downstream proteins in Raw264.7 cells (n = 3). F) qRT-PCR analysis of *Mrc2* expression in Raw264.7 cells incubated with LPS and LPS + PCN. (n = 3). G) The viability of THP-1 macrophages after incubation with various concentrations of RIF

(0-40 μM, for 48 h) with or without LPS/IFNγ (100 ng/mL and 20 ng/mL, for 24 h) (n = 5). H, I) Western blotting and quantification of the protein expression of PXR downstream proteins in THP-1 macrophages (n = 3). THP-1 macrophages were incubated with LPS/IFNγ (100 ng/mL and 20 ng/mL, respectively, for 24 h) with or without RIF (20 μM, for 48 h). Data are expressed as the mean ± SD. $^{\#}P < 0.05$, $^{\#\#}P < 0.01$ and $^{\#\#\#\#}P < 0.0001$ compared with the control group. $^{*}P < 0.05$, $^{**}P < 0.01$, $^{***}P < 0.001$ and $^{****}P < 0.0001$ compared with the LPS or LPS/IFNγ group.

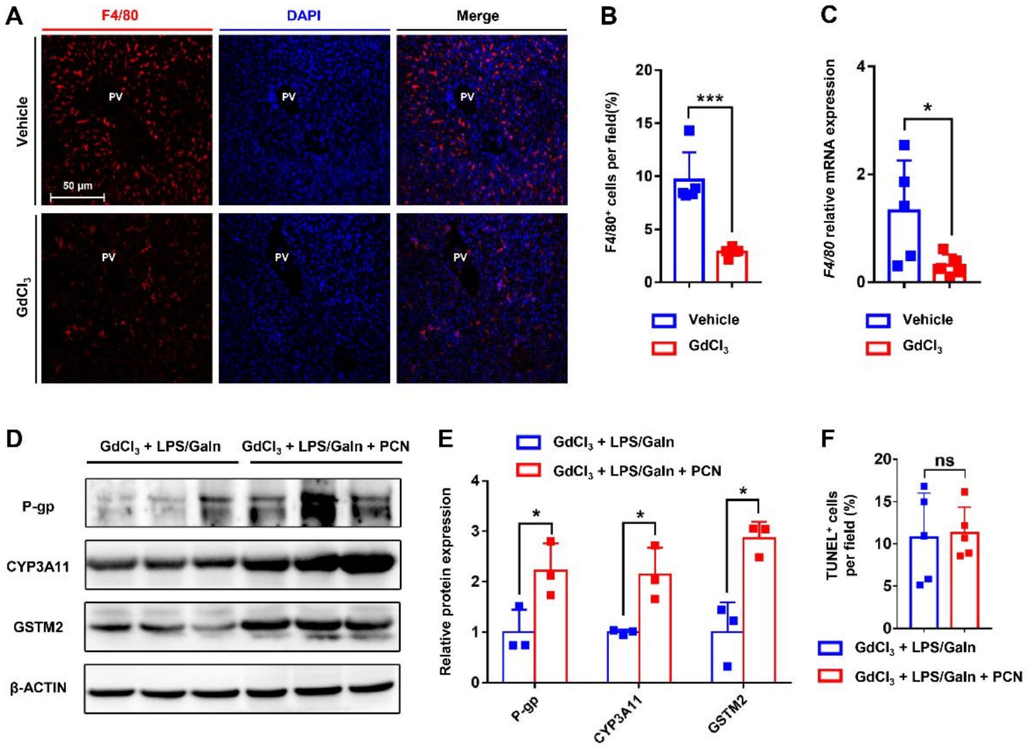

Figure S4. The effects of PXR activation after macrophage deletion by $GdCl_3$ on LPS/GaIn-induced liver injury. A, B) Immunofluorescence staining and quantification of F4/80 (red) in the liver (n = 5). C) qRT-PCR analysis of *F4/80* expression in liver tissue after $GdCl_3$ injection. The data are expressed as the mean ± SD. $^{*}P < 0.05$ and $^{***}P < 0.001$ compared with the vehicle group (n = 5). D, E) Western blotting and quantification of the expression of PXR downstream proteins after PCN treatment and macrophage deletion using $GdCl_3$ (n = 3). F) Quantification of $TUNEL^{+}$ cells of liver sections (n = 5). The data are expressed as the mean ± SD. $^{*}P < 0.05$ compared with the $GdCl_3$ + LPS/GaIn group. $P > 0.05$ compared with the $GdCl_3$ + LPS/GaIn group, ns: not significant.

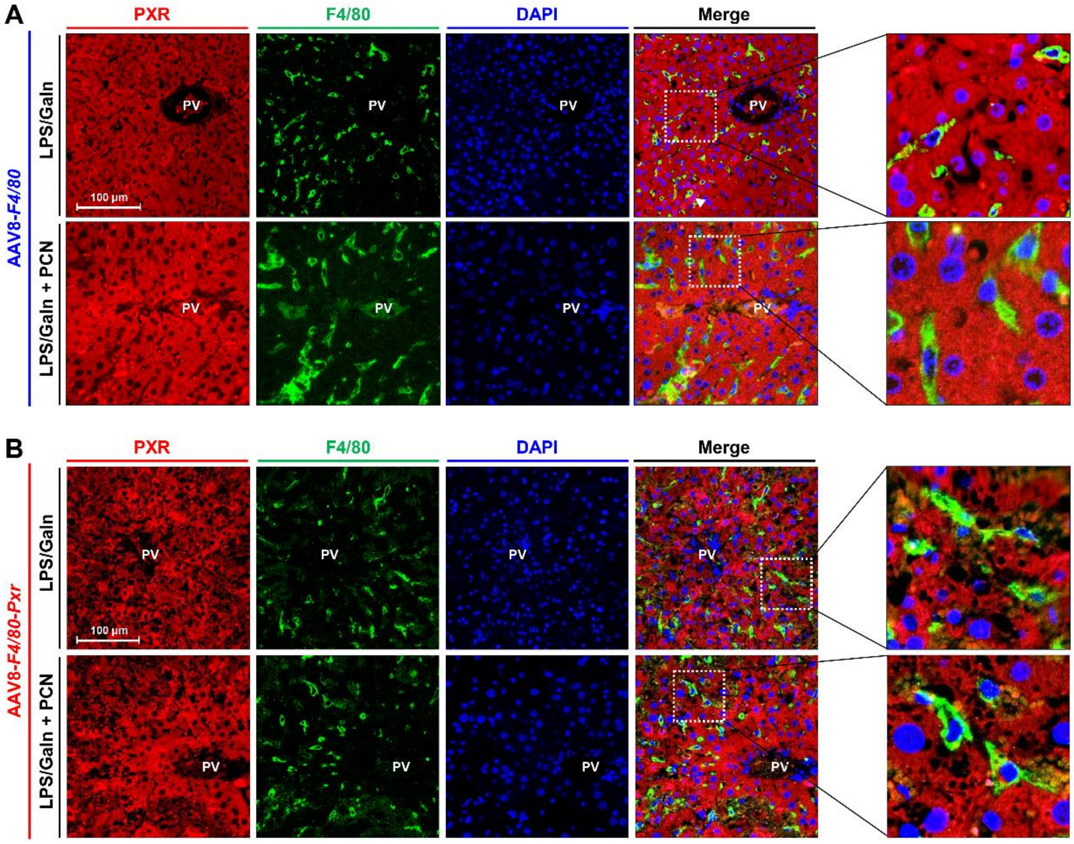

Figure S5. The expression of PXR in AAV-*F4/80*- and AAV-*F4/80-Pxr*-treated mice. A) Immunofluorescence staining of PXR (red) and F4/80 (green) of liver sections of the AAV-*F4/80* group. B) Immunofluorescence staining of PXR (red) and F4/80 (green) of liver sections of the AAV-*F4/80-Pxr* group.

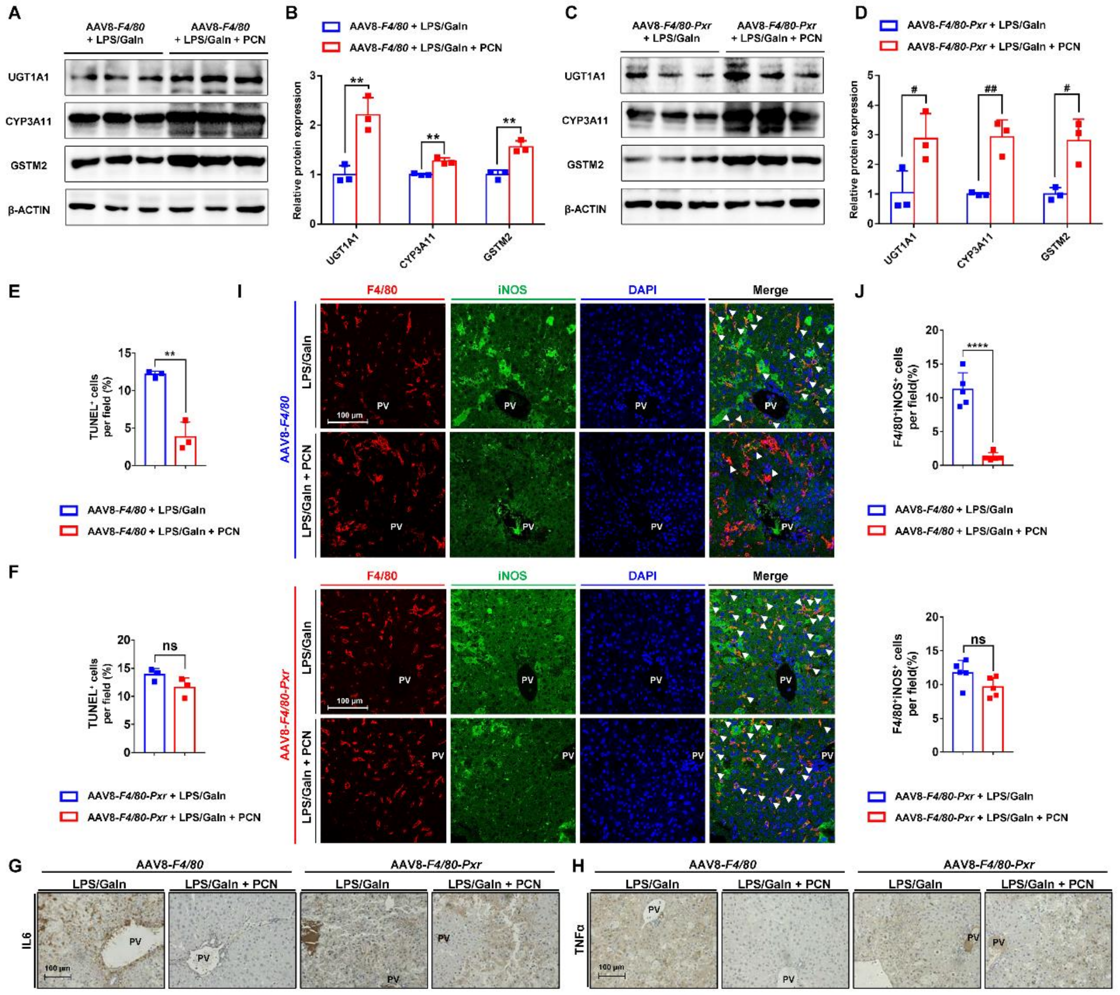

Figure S6. The effects of PXR activation after treatment with AAV8-*F4/80* and AAV8-*F4/80-Pxr*. A-D) Western blotting and quantification of PXR downstream protein expressions (n = 3). E, F) Quantification of TUNEL^{+} cells in liver sections (n = 3). G, H) Immunohistochemical staining of IL6 and TNFα in mice of AAV8-*F4/80* or AAV8-*F4/80-Pxr* groups treated with LPS/GaIn or LPS/GaIn + PCN. I, J) Immunofluorescence staining and quantification of F4/80 (red) and iNOS (green) in mice of AAV8-*F4/80* or AAV8-*F4/80-Pxr* groups treated with LPS/GaIn or LPS/GaIn + PCN (n = 5). Filled triangles indicate the F4/80^{+}iNOS^{+} cells. The data are expressed as the mean ± SD. $^{**}P < 0.01$ and $^{****}P < 0.0001$ compared with the AAV8-*F4/80* + LPS/GaIn group. $^{\#}P < 0.05$ and $^{\#\#}P < 0.01$ compared to the AAV8-*F4/80-Pxr* + LPS/GaIn group. $P > 0.05$ compared with the AAV8-*F4/80-Pxr* + LPS/GaIn group, ns: not significant.

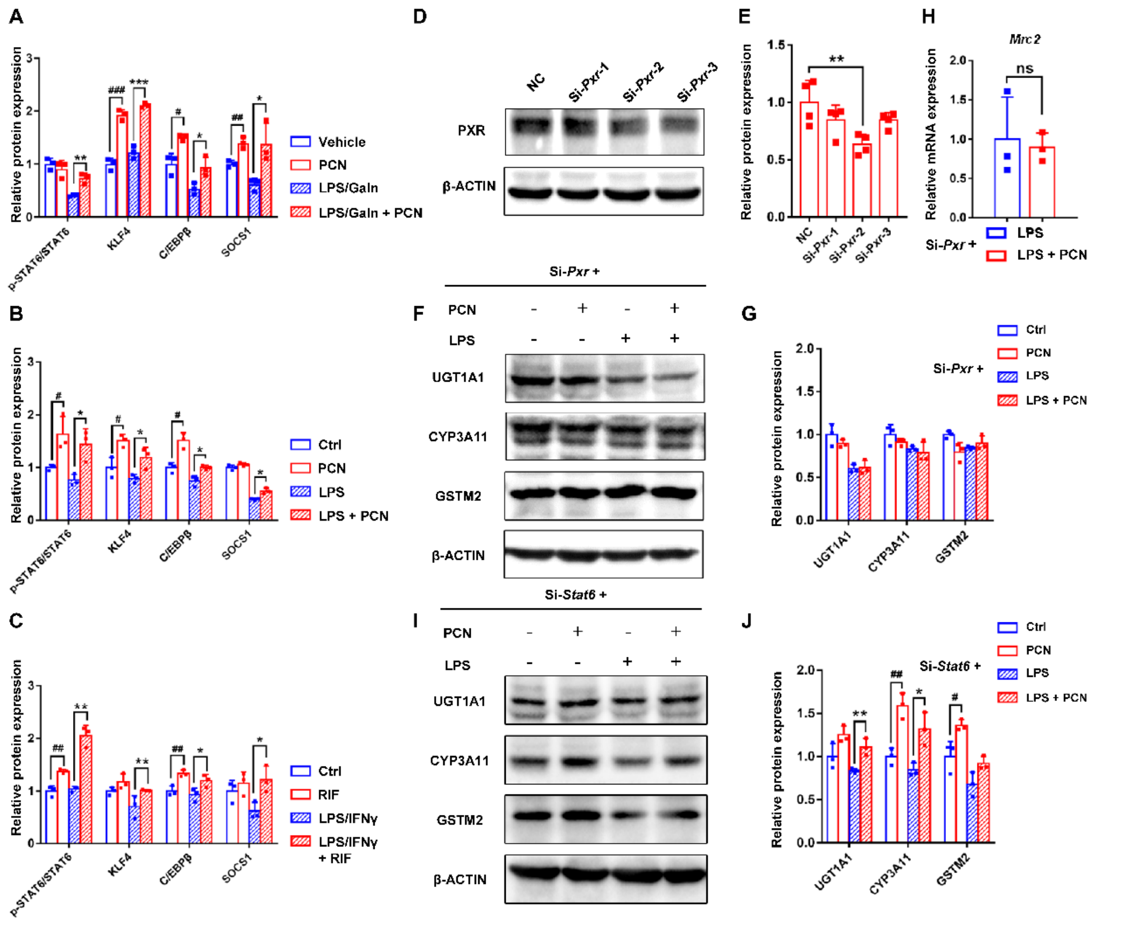

Figure S7. The effects of *Pxr* or *Stat6* silencing on the expressions of the PXR downstream proteins. A) Quantification of p-STAT6/STAT6, KLF4, C/EBPβ and SOCS1 expression in the livers. The data are expressed as the mean ± SD. $^{\#}P < 0.05$, $^{\#\#}P < 0.01$ and $^{\#\#\#}P < 0.001$ compared with the vehicle group. $^{*}P < 0.05$, $^{**}P < 0.01$ and $^{***}P < 0.001$ compared with the LPS/GaIn group. B, C) Quantification of p-STAT6/STAT6, KLF4, C/EBPβ and SOCS1 expression in B) Raw264.7 cells and C) THP-1 macrophages. The data are expressed as the mean ± SD. $^{\#}P < 0.05$ and $^{\#\#}P < 0.01$ compared with the control group. $^{*}P < 0.05$ and $^{**}P < 0.01$ compared with the LPS or LPS/IFNγ group. D, E) Western blotting and quantification analysis of the interference efficiency of si-*Pxr* on PXR protein expression (n = 3). The data are expressed as the mean ± SD. $^{**}P < 0.01$ compared with the negative control group. F, G) Western blotting and quantification of the expressions of PXR downstream proteins after *Pxr* silencing (n = 3). H) qRT-PCR

analysis of *Mrc2* expression in Raw264.7 cells after *Pxr* silencing (n = 3). I, J) Western blotting and quantification of PXR downstream protein expressions after *Stat6* silencing (n = 3). The data are expressed as the mean ± SD. $^{\#}P < 0.05$ and $^{\#\#}P < 0.01$ compared with the control group. $^{*}P < 0.05$ and $^{**}P < 0.01$ compared with the LPS group. $P > 0.05$ compared with the LPS group, ns: not significant.

2. Supplemental tables

Table S1. Sequences for siRNA and shRNA.

SiRNA or shRNA	Sequence (5'-3')
Pxr siRNA-1	GCCGATGTGTCAACCTACA
Pxr siRNA-2	GGAGGAAGATGGAGGTCTT
Pxr siRNA-3	GCGTCATCAACTTCGCCAA
Stat6 siRNA-1	CCAAGACAACAACGCCAAA
Stat6 siRNA-2	GCTGATCATTGGCTTTATT
Stat6 siRNA-3	CCTGCAACCATCTCCTTAT

Table S2. List of mouse primers used in this study.

Gene	Forward (5'-3')	Reverse (5'-3')
Tnfα	AATGGCCTCCCTCTCATCAGTT	CCACTTGGTGGTTTGCTACGA
Nos2	AATCTTGGAGCGAGTTGTGG	CAGGAAGTAGGTGAGGGCTTG
Ccl3	TGAGAGTCTTGGAGGCAGCGA	TGTGGCTACTTGGCAGCAAACA
Arg1	CTCCAAGCCAAAGTCCTTAGAG	AGGAGCTGTCATTAGGGACATC
Mgl1	TGGCCTGAAGCTGACAAGTA	AGGCCGATCCAACTAACCACAT
Fizz1	CCCTTCTCATCTGCATCTCC	CAGTAGCAGTCATCCCAGCA
Mrc2	TGCAAGCAATGCATCCAAGCCT	ACGGCTTTCCGTGTGAGTTT
18s	CCTGGATACCGCAGCTAGGA	GCGGCGCAATACGAATGCCCC

Table S3. List of human primers used in this study.

**Gene**	**Forward (5’-3’)**	**Reverse (5’-3’)**
TNFα	CCTCTCTCTAATCAGCCCTCTG	GAGGACCTGGGAGTAGATGAG
NOS2	TTCAGTATCACAACCTCAGCAAG	TGGACCTGCAAGTTAAAATCCC
CCL2	GATCTCAGTGCAGAGGCTCG	TTTGCTTGTCCAGGTGGTCC
TGM2	CGTGACCAACTACAACTCGG	CATCCACGACTCCACCCAG
MRC1	CTACAAGGGATCGGGTTTATGGA	TTGGCATTGCCTAGTAGCGTA
CCL22	TGGGTGAAGATGATTCTCAATAAGC	CTATAATGGCAGGGAGGTAGGG
18S	ATCCCTGAAAAGTTCCAGCA	CCCTCTTGGTGAGGTCAATG
